# Development and Application of a Novel SPE-Method for Bioassay-Guided Fractionation of Marine Extracts

**DOI:** 10.3390/md13095736

**Published:** 2015-09-11

**Authors:** Adele Cutignano, Genoveffa Nuzzo, Adrianna Ianora, Elvira Luongo, Giovanna Romano, Carmela Gallo, Clementina Sansone, Susanna Aprea, Francesca Mancini, Ugo D’Oro, Angelo Fontana

**Affiliations:** 1 Bio-Organic Chemistry Unit, Institute of Biomolecular Chemistry-CNR, Via Campi Flegrei 34, Pozzuoli, Naples 80078, Italy; 2 Marine Biotechnology Laboratory, Integrative Marine Ecology Department, Stazione Zoologica Anton Dohrn, Villa Comunale, Naples 80121, Italy; 3 GSK Vaccines s.r.l., Via Fiorentina 1, Siena 53100, Italy

**Keywords:** drug discovery, bioactive marine natural products, SPE fractionation methods

## Abstract

The biological diversity of marine habitats is a unique source of chemical compounds with potential use as pharmaceuticals, cosmetics and dietary supplements. However, biological screening and chemical analysis of marine extracts pose specific technical constraints and require adequate sample preparation. Here we report an improved method on Solid Phase Extraction (SPE) to fractionate organic extracts containing high concentration of salt that hampers the recovery of secondary metabolites. The procedure uses a water suspension to load the extracts on a poly(styrene-divynylbenzene)-based support and a stepwise organic solvent elution to effectively desalt and fractionate the organic components. The novel protocol has been tested on MeOH-soluble material from three model organisms (*Reniera sarai*, *Dendrilla membranosa* and *Amphidinium carterae*) and was validated on a small panel of 47 marine samples, including sponges and protists, within discovery programs for identification of immuno-stimulatory and anti-infective natural products.

## 1. Introduction

Marine natural products (MNPs) continue to be a source of inspiration in many areas of biomedical science. Due to the physical and chemical properties of the marine environment, almost all classes of marine organisms exhibit a diversity of molecules with unique structural features [1]. Yet, it has been recently estimated that between 700,000 and one million species live in the world’s oceans [2], thus the species so far investigated represent only a small percentage of the total number of the marine existing organisms.

The major effort in MNPs discovery is focused on compounds of pharmaceutical interest with an increasing number of molecules for drug development having been reported in recent years [3]. Identification of bioactive chemicals is a complex task that requires multidisciplinary interactions. Continuous upgrade of analytical and molecular techniques is important in this process and is a prerequisite to target novel products by high-throughput approaches.

Solid phase extraction (SPE) has become a common technique for isolation, concentration, clean-up and medium exchange. In relation to MNPs discovery, the versatility of SPE is widely applied as a pre-fractionation step [4,5,6] to address many purposes, including purification, trace enrichment, desalting, derivatisation and class fractionation. Furthermore, access to more selective and easy-to-use phases allows to simplify the analytical procedures and to reduce the amount of sample to deal with.

The aim of this work was to develop an original SPE-based method to select and effectively fractionate marine natural extracts in order to obtain in one step a rough separation of the main major chemical classes. The analytical protocol was also designed to be integrated in traditional procedures of high-throughput bioassay guided fractionation.

## 2. Results and Discussion

Sample manipulation can significantly affect the reliability of a bioassay guided fractionation. The process, which has to cover the range of polarity of the metabolites in the sample, allows recovery of minor components in enriched fractions, the activity of which may be masked in the raw extract. In marine samples, this is the most critical issue, since the overwhelming presence of salts can significantly lead to overestimation of the extract weight and misinterpretation of the hypothetical level of the bioactive components. To address this aim, extracts of marine samples have been traditionally partitioned with organic solvents of increasing polarity in order to enrich the amount of secondary metabolites recovered and reduce the masking effect of salt. Independently of the efficiency, this procedure can be highly time consuming, solvent demanding, poorly reproducible and expensive. Since the pioneering applications in early 80s, materials based on poly(styrene-divinylbenzene) (PS-DVB) have been largely used as chromatographic support for fractionation and purification of organic components from complex matrices [7,8,9]. In particular, PS-DVB resin is an attractive adsorbent for extraction of various types of organic compounds due to pH stability and the hydrophobic surfaces that highly retain non-polar molecules. For these properties, different commercial types of PS-DVB absorbent (e.g., Amberlite^®^ XAD^®^-2, Diaion^®^ HP-20, Sigma-Aldrich, Milan, Italy) have been long used by independent authors to desalt and fractionate marine extracts as non-exhaustive examples, see [10,11,12,13,14]. More recently, the potential of PS-DVB (Diaion^®^ HP-20SS, Sigma-Aldrich, Milan, Italy) as an alternative to prepare purified natural product libraries has been carefully evaluated by Nagle and co-workers [15]. By using selected plant extracts as test panel, these authors concluded that cross-linked polystyrene matrix retained considerable bioactivities and reduced loss of bioactive natural products in comparison with normal-phase sorbents.

Starting from this consideration, we explored the use of a spherical PS-DVB resin for SPE (Chromabond^®^ HR-X, Düren, Germany) together with an alternative stepwise elution in order to desalt the samples and to obtain a reproducible resolution of the main chemical classes of common marine extracts. In comparison with other PS-DVB resins (e.g., Diaion^®^ HP-20, Sigma-Aldrich, Milan, Italy) that have been used with the same aim [11,13], Chromabond^®^ HR-X offers the advantage to have narrow pores (260 Å) and regular, little particles (around 65 μm) that increase the surface area.

After a careful analysis of the material that was available in our laboratory, three organisms containing chemical known products that both represent common marine metabolites and cover a wide range of polarity were selected to test the procedure (Table 1).

**Table 1:** Marine biological samples evaluated as test panel and the corresponding known bioactive constituents.

Phylum	Sample Name	Main Secondary Component		Ref.
Porifera	*Dendrillamembranosa*	9,11-dihydrogracilin A	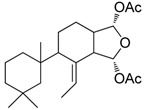 **1**	[16]
Porifera	*Renierasarai*	sarains A–C	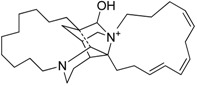 e.g., sarain A (**2**)	[17,18]
		sarains 1–3	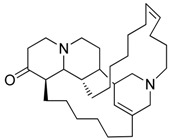 e.g., sarain 1 (**3**)	
Dinoflagellata	*Amphidiniumcarterae* (CCMP121)		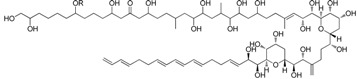	[19]
		amphidinol-18amphidinol-19	**4** R = H **5** R = SO_3_^−^	

The methanol extracts of these organisms were dried at reduced pressure and re-suspended in a minimal volume (1 mL) of distilled water. The aqueous suspensions were then loaded onto the pre-packed CHROMABOND^®^ HR-X cartridges (6 mL/500 mg) and submitted to a five-step elution protocol. After a preliminary desalting step with 2 mL of distilled water, fractionation of the organic components was achieved by elution with 100% H_2_O (fraction A, 18 mL) followed by solvent mixtures of increasing chromatographic strength from CH_3_OH/H_2_O (fraction B, 50:50, 24 mL) to CH_3_CN/H_2_O (fraction C, 70:30, 18 mL), 100% CH_3_CN (fraction D, 18 mL) and, finally, CH_2_Cl_2_/CH_3_OH (fraction E, 90:10, 18 mL). The polarity sequence of these eluents gave good recovery with a fair distribution of the major components in the five fractions. Interestingly, primary metabolites (mainly, sugars, amino acids, sterols, fats, phospholipids, triglycerides) showed a predictable chromatographic behavior using TLC analysis (see Experimental Section 3.3), being selectively eluted as follows: saccharides and amino acids in fraction A, nucleosides in B, polar lipids including glycolipids and most phospholipids in C, sterols and free fatty acids in D and, finally, triglycerides and other neutral lipids in E. Analogously, the different classes of secondary metabolites occurring in the selected organisms also distributed widely among the five fractions. Thus, *D. membranosa* afforded a unique fraction (fraction D) containing 9,11-dihydrogracilin A (**1**), whereas the polyhydroxylated metabolites of *A. carterae* (Amphidinols **4** and **5**) were concentrated in fraction C. Fractionation of *R. sarai* extracts gave more complex results. The sponge alkaloids were nicely distributed in two fractions according to the heterogeneous chemistry of these typical marine metabolites. Thus, caged zwitterionic compounds of the series of sarains A–C (e.g., **2**) were eluted by CH_3_CN/H_2_O 70:30 whereas the less complex sarains 1–3 (e.g., **3**) and the corresponding *iso*-sarains1–3 were collected by MeOH/H_2_O 50:50. The LC-MS analysis of these fractions also revealed the presence of a number of minor analogs that have been not reported before. The characterization of these molecules is beyond the scope of the present work but their occurrence underlines the potential of the SPE method here presented. In fact, purification of sarains as accomplished by Cimino and coworkers [17,18] entailed a complex and time-demanding chromatographic procedure that was not free of material loss. In line with our working hypothesis, the SPE method overcame most of these issues and furthermore offered the advantages of removing the interfering components and revealing the presence of minor compounds.

**Figure 1:**
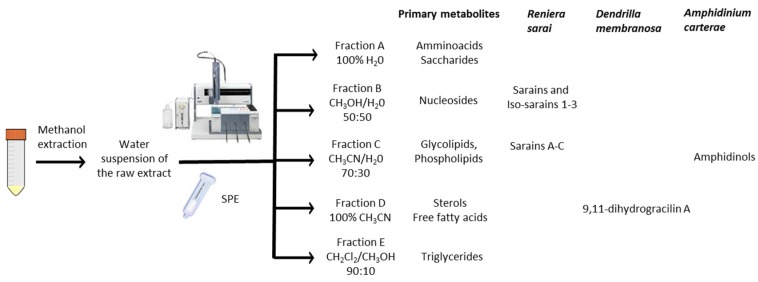
Schematic outline of the fractionation process by the automated Solid Phase Extraction (SPE) procedure.

The weights of the combined fractions, were used to calculate total sample recovery from both sponge and dinoflagellate extracts. Including the salt from the wash step, the procedure gave above 80% recovery over three replicates of each sample (87% ± 6% with *D. membranosa*; 83% ± 3% with *R. sarai*; 84% ± 5% with *A. carterae*). Once the reliability of the elution protocol was established, we transferred the method onto a completely automated fractionation system in order to validate the procedure for high-throughput studies (Figure 1). The use of a branded analytical platform for loading and eluting the sample on pre-packed cartridges required only an adjustment of the solvent volumes but did not entail changes in the elution order (Table 2).

**Table 2:** Fractionation protocol of marine extracts on SPE/HR-X column (6 mL/500 mg) by using automated stepwise elution.

(1) Sample Preparation	(2) Column Equilibration	(3) Elution Gradient:
• Add 1 mL of H_2_O; • Sonicate.	• 3 mL CH_3_OH; • 6 mL H_2_O.	W—Washing step 100% H_2_O (2 mL); 100% H_2_O (6 mL); CH_3_OH/H_2_O (50:50, 9 mL); CH_3_CN/H_2_O (70:30, 9 mL); 100% CH_3_CN (9 mL); CH_2_Cl_2_/CH_3_OH (90:10, 9 mL).

Considering the desired application of the method for drug-discovery, the first aim was to establish the amount for each fraction to achieve both the first steps of a bioassay-guided screening and a preliminary chemical characterization of the products by LC-MS and NMR. Different amounts of extracts were submitted to the analytical procedure in a range between 10 and 50 mg since for each organic matrix the optimal quantity of sample has to be established based on the salt content. As expected, automation gave the possibility to quickly process large numbers of samples in a short time thus simplifying the preparation of a natural products library. This was proven by application of the protocol for screening 47 samples including 27 microalgae assayed for immunomodulatory activity and 20 samples of microalgae and Antarctic sponges assessed for antimicrobial properties. Each sample (20 mg for microalgae and 40 mg for sponges) was processed in less than one hour and the whole batch of samples required almost two days of continuous work.

**Figure 2:**
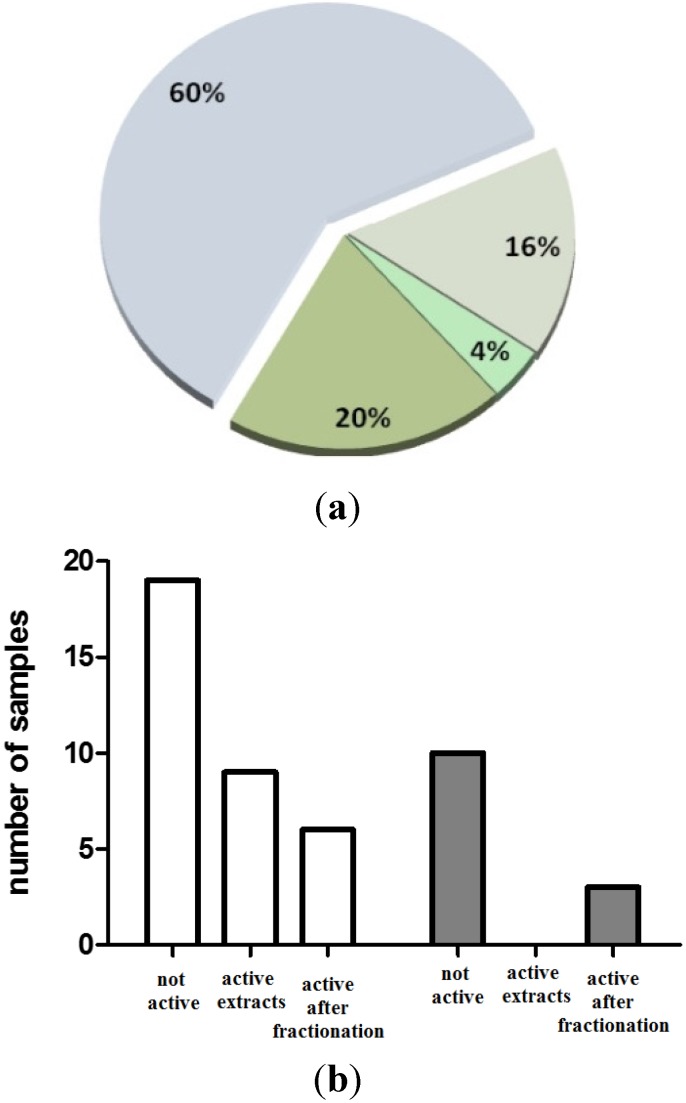
Score hit over 47 marine samples tested for immunomodulatory and antimicrobial activity. (**a**) Response is reported as negative extracts (60%, pale blue), positive extracts (16%, pale green), positive extracts loosing activity after fractionation (4%, green) and positive hit only after fractionation (20%, dark green); (**b**) distribution of the activity within protists (*n* = 34) (white) and sponges (*n* = 13) (grey).

The analysis of all the tested samples (extracts and SPE-fractions) gave 40% of positive hits with the interesting result that 50% of the activity was detectable only after extract fractionation (Figure 2). In fact, SPE fractions contained less salt (mainly NaCl) than primary extracts, as measured according to the method proposed by Zhu and Lee [20]. Consequently, the relative concentrations of the active products were enhanced in these samples, thus increasing the chances of positive response in the biological screening. This was marked with extracts of sponges that generally contained higher level of NaCl and probably other interfering components than those of protists. Furthermore, except for two samples (see below), we observed a general increase in activity of the fractions in comparison to extracts.

More specifically, immunostimulatory activity was estimated as the ability to trigger response by immune cells [21,22]. Specific activation was measured by release of interleukin-6 (IL-6) from human peripheral blood mononuclear cells (PBMCs). Activity was expressed as percentage of IL-6 induced by natural fractions in comparison with the maximal effect induced by PAM_3_CSK_4_ (100% response), a synthetic triacylated lipopeptide that mimics the acylated amino terminus of bacterial lipoproteins and triggers the hetero-dimeric receptor composed of Toll like receptor (TLR) 1 and TLR2 [23]. Data analysis of the SPE protocol applied to evaluation of algal lipids as immunostimulants gave 44% positive hits over 27 tested samples (4% of the maximal production of IL-6). For eight of these samples, statistical analysis by Pearson’s correlation between control (PAM_3_CSK_4_) and test groups identified a significantly increased activity with PBMC response after fractionation.

**Figure 3:**
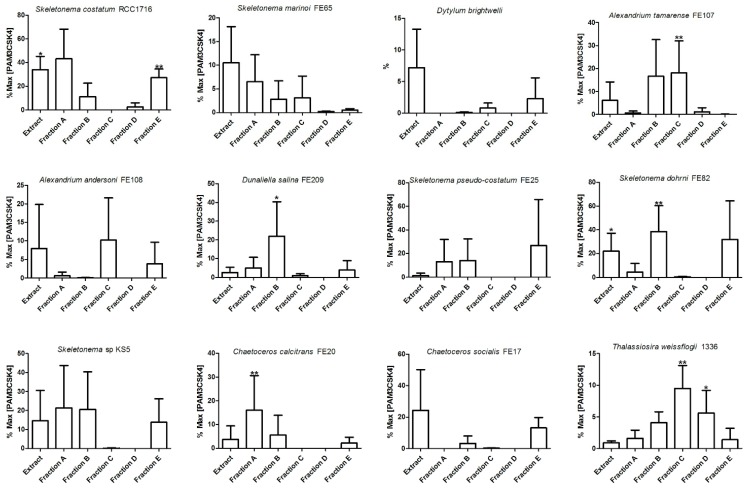
Activity of microalgal samples (extracts and A–E fractions) tested for IL-6 production in human peripheral blood mononuclear cells (PBMCs). Activity is expressed as percent of maximal response induced by PAM_3_CSK_4_, a synthetic lipopeptide agonist of TLR1/2. Data are presented as mean value of 3 replicates and analysed through Pearson’s correlation. Variations were considered significant (*) with 0 < *p*-value ≤ 0.3 while strongly significant (**) with *p* > 0.7.

It is worth noting that the high variability of the data is basically due the different response of the PBMC donors. Study on these extracts are currently in progress but, from a chemical point of view, it is to note that the activity was distributed among different fractions, thus suggesting the presence of different chemical effectors (Figure 3). In two cases (*Skeletonema marinoi* FE65 and *Chaetoceros socialis* FE17) we observed loss of activity after fractionation likely due to inherent chemical instability of the active components (Figure 3).

**Figure 4:**
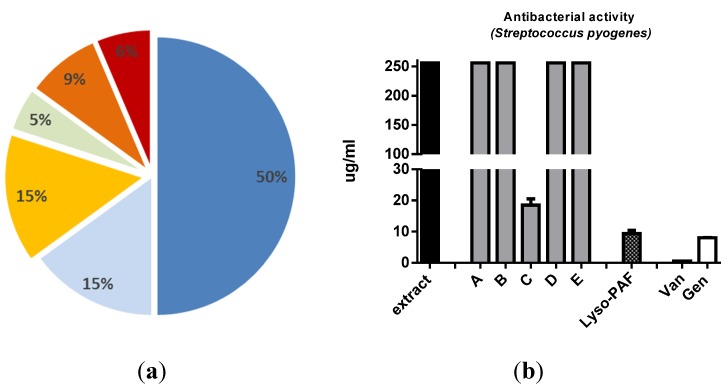
Score hit over 20 marine samples tested for antimicrobial activity. (**a**) Response distribution between sponges and protists: inactive sponge extracts (50%, blue), inactive protist extracts (15%, pale blue), antibacterial activity from sponges only after fractionation (15%, orange), antifungal (*Candida albicans*) protist extracts (5%, green), antibacterial activity from protists only after fractionation (9%, dark orange), antifungal activity from protists only after fractionation (6%, red); (**b**) increase of antibacterial activity during fractionation of sponge extracts. MIC (μg/mL) of the Antarctic sponge extract ICB-BA99, its corresponding SPE/HR-X fractions (A–E) and the pool of LysoPAF analogs **6a**–**6h** against *Streptococcus pyogenes* (L49). Van = Vancomycin; Gen = Gentamicyn.

For the second application, the extraction protocol was used to screen 13 Antarctic sponges and seven selected protists (three dinoflagellates, three diatoms and one green microalga) for identification of potential anti-infective candidates. After fractionation on SPE/HR-X, the small group of samples was tested against both the pathogenic fungus *Candida albicans* and a panel of infective bacteria (*Staphylococcus aureus MSSA*, *Streptococcus pyogenes*, *Enterococcus faecium*, *Moraxella catharralis*, wild and hyperpermeable strains of *Escherichia coli*) at concentrations ranging from 1 to 256 μg/mL for both extracts and SPE-fractions. One third of the samples showed antimicrobial activity in the range from 3 to 64 μg/mL (Figure 4a).

**Figure 5:**
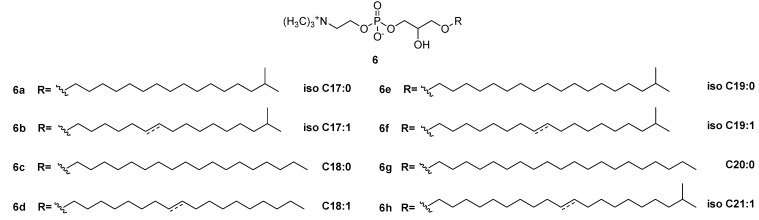
Structures of Lyso-PAF analogs **6a**–**6h** from the Antarctic spongeICB-BA99.

In agreement with the literature [24,25], antifungal activity was detected in the dinoflagellate *Amphidinium carterae* (CCMP121) containing Amphidinol-18 (**4**), whereas another species of this genus, namely *Amphidinium massartii* (ICB-BCL19), revealed inhibitory activity (Minimum Inhibitory Concentration, MIC) against both groups of microorganisms (study in progress). Except for *A. carterae*, the anti-infective activity of these samples was detectable only after fractionation by SPE, thus further proving the reliability of the novel protocol. The three samples of Antarctic sponges (ICB-BA83, ICB-BA98 and ICB-BA99) revealed mild comparable activity against *Streptococcus pyogenes* (MIC 64, 37.5 and 18 μg/mL, respectively) associated to the fraction eluted by CH_3_CN/H_2_O. To verify the finding, the material from the most active species ICB-BA99 was processed according to standard bioassay-guided fractionation to give a mixture of lysoplatelet-activating factors (Lyso-PAF), **(**Figure 5) with MIC ranging between 12.5 and 9.4 μg/mL. Analogous fractionation of the other two species gave similar activity (results not shown). The antimicrobial activity of Lyso-PAF from marine sponges has been already reported [26,27,28,29,30] and is comparable to the activity found for the analogs **6a**–**6h** (Figure 4b).

## 3. Experimental Section

### 3.1. General

NMR spectra were recorded on Bruker DRX 600 spectrometer equipped with an inverse TCI CryoProbe. Chemical shifts values are reported in ppm (δ) and referenced to internal signals of residual protons (DMSO-*d*_6_, ^1^H δ 2.50). High resolution mass spectra were acquired on a Q-Exactive Hybrid Quadrupole-Orbitrap Mass Spectrometer (Thermo Scientific, Milan, Italy). HPLC analyses were performed on a Jasco system (PU-2089 Plus-Quaternary Gradient Pump equipped with a Jasco MD-2018 Plus Photodiode Array Detector (Jasco, Cremella, Italy) and ELSD detector (SEDEX85, SEDERE LT-ELSD). For centrifugation was used an Allegra X-12R centrifuge (Beckman Coulter, Milan, Italy). Solid Phase Extraction (SPE) was carried out using both prepacked and not polystyrene-divinyl benzene columns (CHROMABOND^®^ HR-X, Macherey-Nagel, Düren, Germany). Automated fractionations were carried out on GX-271 ASPEC Gilson apparatus equipped with a TRILUTION^®^ LH Software (Gilson, Middleton, WI, USA). Silica gel chromatography was performed using precoated Merck F254 plates. All the chemicals and solvents (Sigma-Aldrich, Milan, Italy) were of analytical reagent grade and were used without any further purification.

### 3.2. Algal Culturing

The marine microalgae are from the phytoplankton culture collection of the Stazione Zoologica Anton Dohrn (Naples, Italy). Microalgae were cultured in 10 L carboys filled with 0.22 μm FSW enriched with Keller (*K*) medium [31] for flagellates and dinoflagellate species or with *f*/*2* medium [32] for diatoms. Cultures were gently bubbled with filtered ambient air and were grown in a climate chamber (RefCon, Naples, Italy) at 20 °C under 12 h:12 h light:dark cycle (100 μmol photons·m^2^·s^−1^). Cells were harvested at the stationary phase by centrifugation at 3750 rpm for 10 min at 4 °C in a swing-out rotor (DR 15P, Braun Biotechnology International, Allentown, PA, USA).

### 3.3. Extraction and Fractionation

Humid microalgal cell pellets (*ca*. 200 mg) were suspended in MeOH (1:5, *w*/*v*) and extracted by sonication; after centrifugation at 4000 rpm for 5 min at room temperature, the organic phase was recovered and dried at reduced pressure. For marine invertebrates the frozen specimen were lyophilized and about 300 mg of dry material was extracted with MeOH (100 mL). After sonication, the organic phase was decanted and dried under vacuum. The extracts were stored at −80 °C until use.

Fractionation of each extract (about 20 mg for microalgae and 40 mg for sponges) was achieved on a GX-271 ASPEC Gilson apparatus by using CHROMABOND^®^ HR-X cartridges (6 mL/500 mg). The cartridge was conditioned with 3 mL of methanol and equilibrated with 6 mL of distilled water. The extract was suspended in 1 mL of distilled water and sonicated for a few seconds in an ultrasonic bath before loading onto the column. Elution steps are reported in Table 2.

SPE fractions were analyzed by TLC developed with petroleum ether/diethyl ether (60:40, *v*/*v*), CHCl_3_:CH_3_OH (95:5, *v*/*v*) and CHCl_3_:CH_3_OH:H_2_O (65:25:4, *v*/*v*/*v*) and revealed by spraying with Ce(SO_4_)_2_. Furthermore, for each fraction a ^1^H-NMR spectrum in DMSO-*d*_6_ and a LC-MS profile (Phenomenex Luna-C18(2) column 5 μm, 150 mm × 2.1 mm, with a linear gradient of CH_3_OH/H_2_O from 60% of CH_3_OH to 100% in 40 min, flow 0.2 mL/min) were recorded for dereplication purposes.

### 3.4. Isolation and Characterization of Lyso-PAF Analogs from Antarctic Sponges

Each sponge extract (1 g) was fractionated on open bed column (2 cm diameter) packed with HR-X resin (2.5 g) eluting with the developed protocol affording about 20 mg of bioactive fraction C eluted with CH_3_CN/H_2_O. HPLC purification of the metabolites was performed on a Luna Phenyl-hexyl column (Phenomenex, 5 μm, 250 mm × 4.6 mm) with a gradient of CH_3_OH/H_2_O from 80% to 90% CH_3_OH in 10 min and then to 100% CH_3_OH in 20 min, with a flow of 1 mL/min monitoring by both ELSD and PDA detectors. The active compounds were collected in the time range between 10 and 15 min. The identification of lyso-PAF congeners (Figure 5) was accomplished by NMR analysis in CD_3_OD and by comparison of spectroscopic and spectrometric data with those reported in the literature [26,27,28,29,30].

### 3.5. Antibacterial Test

Strain of *Staphylococcus aureus* (MSSA), *Streptococcus pyogenes*, *Enterococcus faecium*, *Moraxella catharralis*, wild and hyperpermeable strains of *Escherichia coli* were used in the antibacterial tests. The bacteria were grown overnight, diluted 1:1000 in 10 mM sodium phosphate buffer (pH 7.5), and incubated with increasing concentrations of different compounds at a density of 4000 colony forming units (CFUs) per milliliter. After 4 h at 37 °C, serial dilutions of each protein-bacteria mix were prepared and plated on chloramphenicol (5 μg/mL) containing medium, and colonies formed after each treatment were determined.

### 3.6. Immunostimulatory Test

Immunostimulating activity was evaluated as the ability to induce release of interleukin-6 (IL-6) from human peripheral blood mononuclear cells (PBMCs). Human PBMCs were isolated from buffy coats of healthy donors using Ficoll gradient (Amersham Biosciences, Buckinghamshire, UK) and cultured in RPMI 1640 (GIBCO) supplemented with 100 U/mL penicillin, 100 μg/mL streptomycin and 2 mM glutamine(all from GIBCO), and 10% heat-inactivated Fetal calf serum (Euroclone, Milan, Italy). Buffy coats from healthy donors were obtained from the Blood Transfusion Section, Empoli Hospital. Informed consent was obtained before all blood donations. The study protocol was approved by the Empoli Hospital ethical committee and conforms to the ethical guidelines of the 1975 Declaration of Helsinki. PBMCs were seeded in 96-well round bottom plates (1 × 10^5^ cells/well) and stimulated over night with different concentrations of microalgal extracts. Supernatants were collected and IL-6 concentration was measured by standard ELISA. For each samples, the maximum efficacy of activation was expressed as the percentage of maximum activation elicited by the benchmark compound PAM_3_CSK_4_.

### 3.7. Statistical Analysis

Statistical calculations were performed using Microsoft Excel for Windows software (version 3.6.5, Microsoft Office). Values after treatment within each group were analyzed using paired Student’s *t*-test (*p* ≤ 0.05). Variations of the predictive value (about 20%) for the key response parameter of interleukin-6 (IL-6) release from human peripheral blood mononuclear cells (PBMCs) were measured by Pearson’s correlations between control and test groups. All data are presented as mean value and data analyzed through Pearson’s correlation were considered significant (*) with 0 < *p*-value ≤ 0.3 while strongly significant (**) with *p* > 0.7.

## 4. Conclusions

Marine organisms are an important source of bioactive natural products with seven molecules approved for human use in the latest decade [33]. While a definitive picture is not possible, Mayer reported that more than two hundred novel compounds have been indicated of pharmacological interest by preclinical assays only in the 3 year period from 2009 to 2011 [34]. This suggests that the field is far from being fully explored. Nevertheless, identification of marine bioactive substances faces a number of technical challenges that include variability of the source material and the difficulty of isolating the active constituents. In particular, crude extracts of marine organisms pose a dual problem. Marine samples are complex mixtures, containing hundreds of compounds, overwhelmed by salts. High concentrations of salts are toxic for many cells so that crude marine material cannot be directly tested. Although this problem is solvable in principle by desalting procedures, the process must avoid loss of active compounds and, if possible, allow bioactivity-directed fractionation. Here we have described a novel procedure for fractionation of marine extracts by polystyrene-divinyl benzene pre-packed columns (CHROMABOND^®^ HR-X) and an automated system of elution (GX-271 ASPEC Gilson apparatus) that uses five different solvents to achieve salt removal and a predictable distribution of metabolites.

The procedure allowed good separation of terpene 1, alkaloids 2–3 and polyketides 4–5, which were selected to represent the most common classes of metabolites occurring in extracts of marine organisms [16,17,18,19]. The use of the present method was particularly effective in extraction and fractionation of sarains, the complex alkaloids of the Mediterranean sponge *R. sarai*. Alkaloids are one of the most important class of natural products and a major inspiration of drugs since ancient times. Isolation of complex alkaloids from marine extracts is always problematic because polarity of these compounds reduces the effectiveness of traditional approaches based on liquid-liquid extraction. The new method offers exciting opportunities to avoid or at least ameliorate many of the difficulties associated with screening of marine natural extracts [35]. Since extracts can be loaded as suspension onto the pre-packed column, the procedure can be used to simply desalt and fractionate crude material. Otherwise it can be aimed at creating “peak libraries” in which crude extracts are pre-fractionated into a series of enriched fractions which can be rapidly characterized by spectroscopic and spectrometric methods, as well as screened by HTS.

## Supplementary Files

Supplementary File 1
