# Supplementary material for: Development and Application of a Novel SPE-Method for Bioassay-Guided Fractionation of Marine Extracts
Source: Mar Drugs. 2015 Sep 11;13(9):5736–49. doi: 10.3390/md13095736 (PMC4584351; doi:10.3390/md13095736)
Supplement: Supplementary File 1 [file marinedrugs-13-05736-s001.docx]

Supporting Information


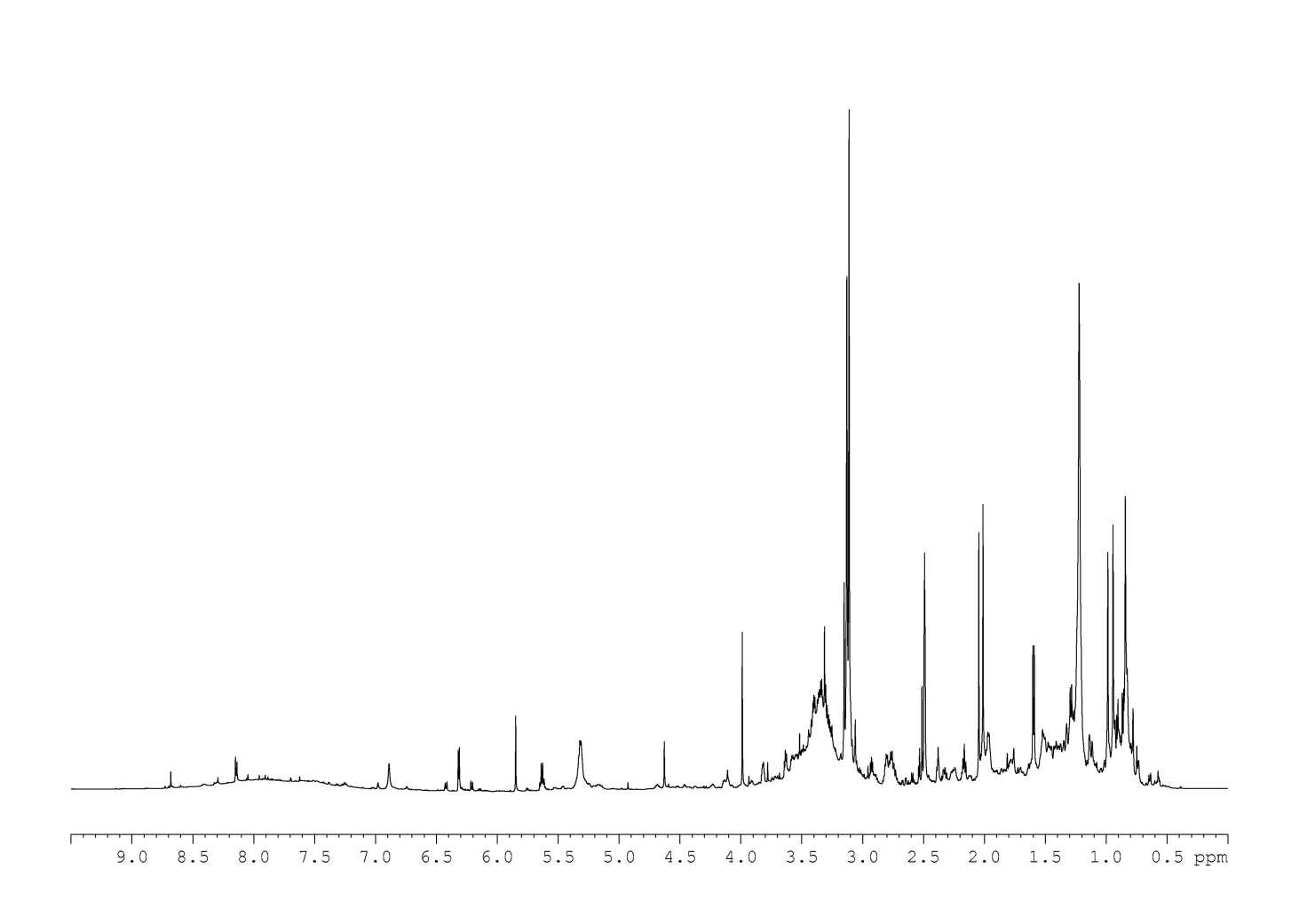


**Figure S1.** ^1^H-NMR spectrum of *Dendrilla membranosa* extract (600 MHz, DMSO-*d*_6_).


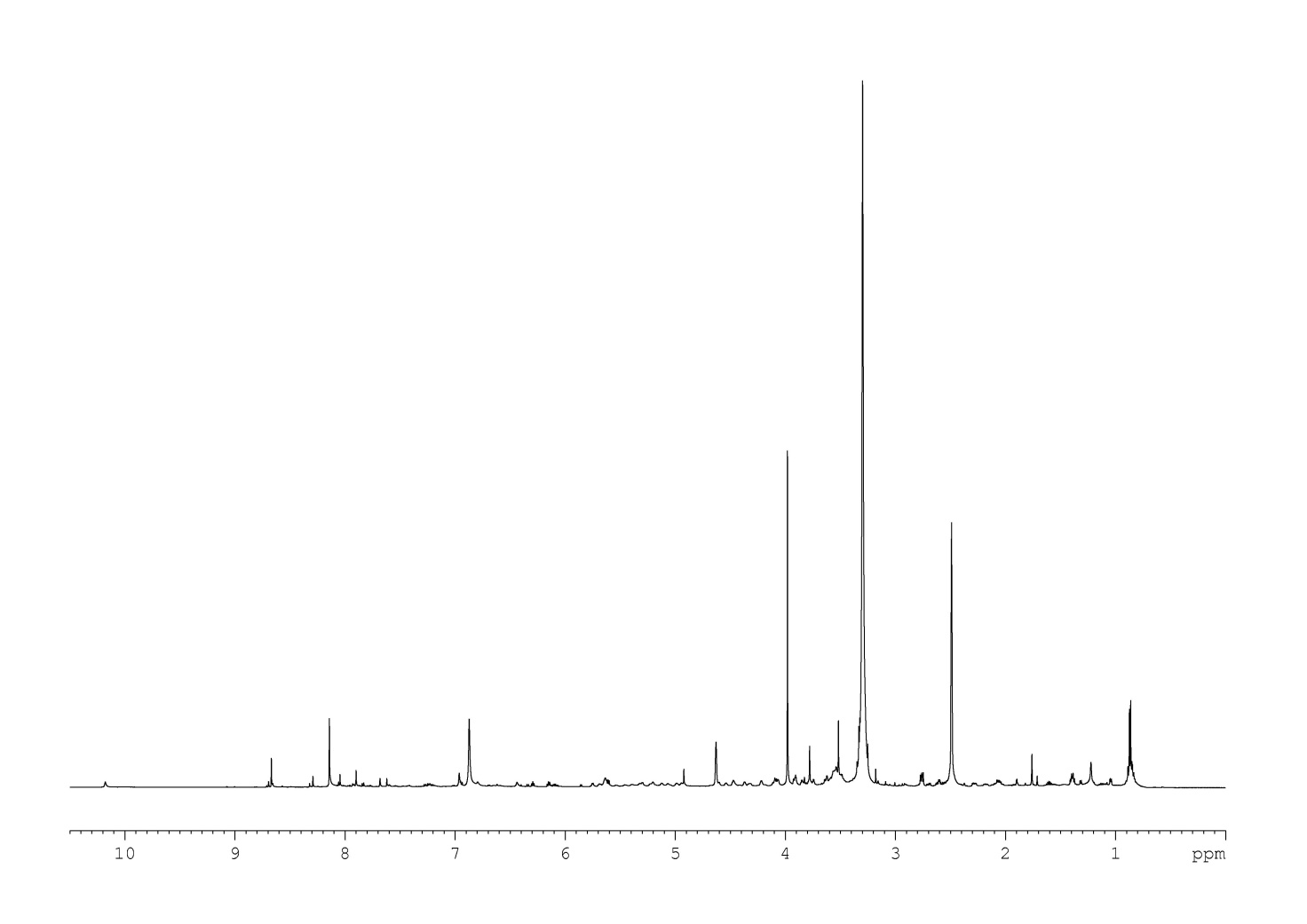


**Figure S2.** ^1^H-NMR spectrum of *Dendrilla membranosa* SPE-fraction B (600 MHz,
DMSO-*d*_6_).


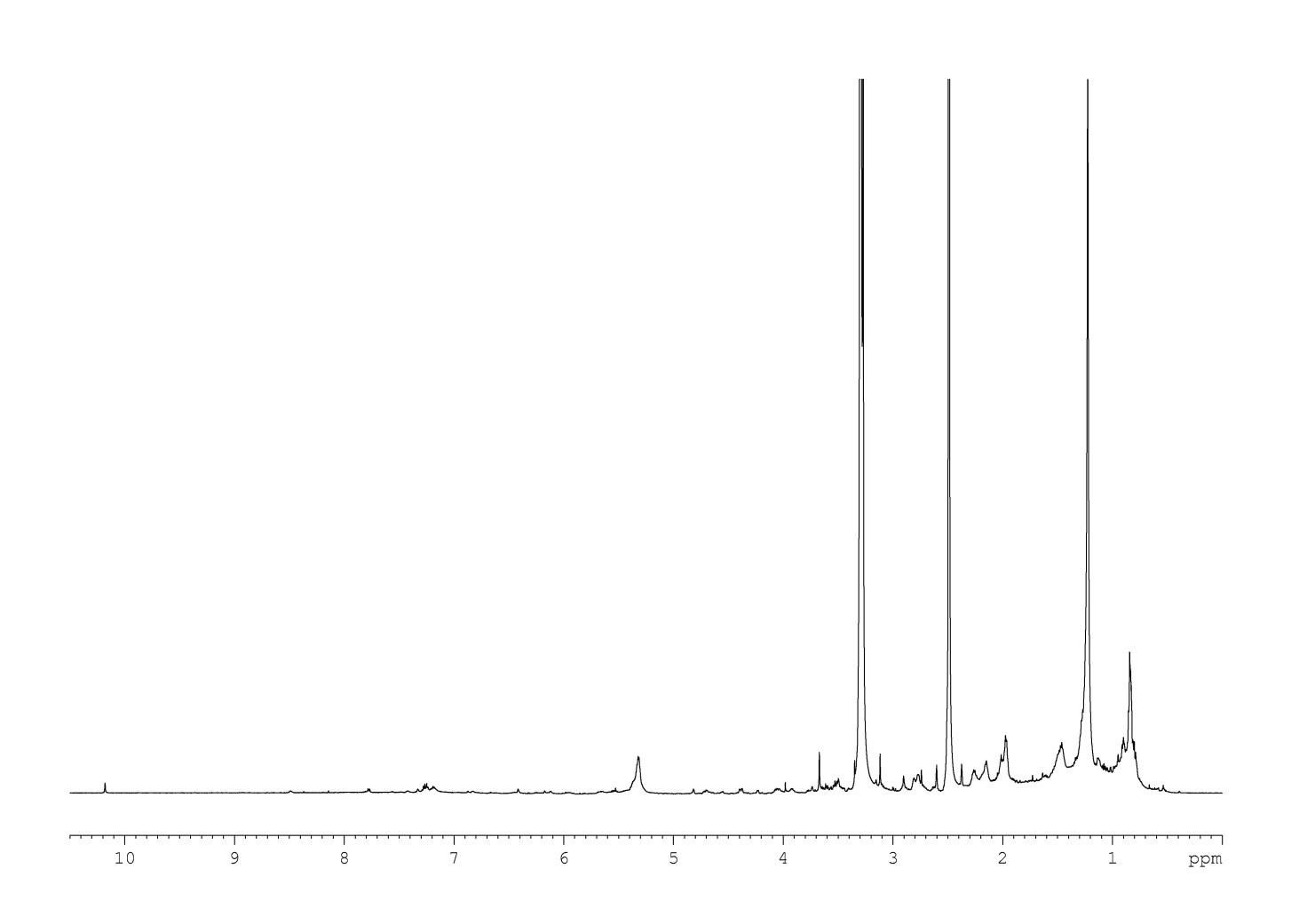


**Figure S3.** ^1^H-NMR spectrum of *Dendrilla membranosa* SPE-fraction C (600 MHz,
DMSO-*d*_6_).


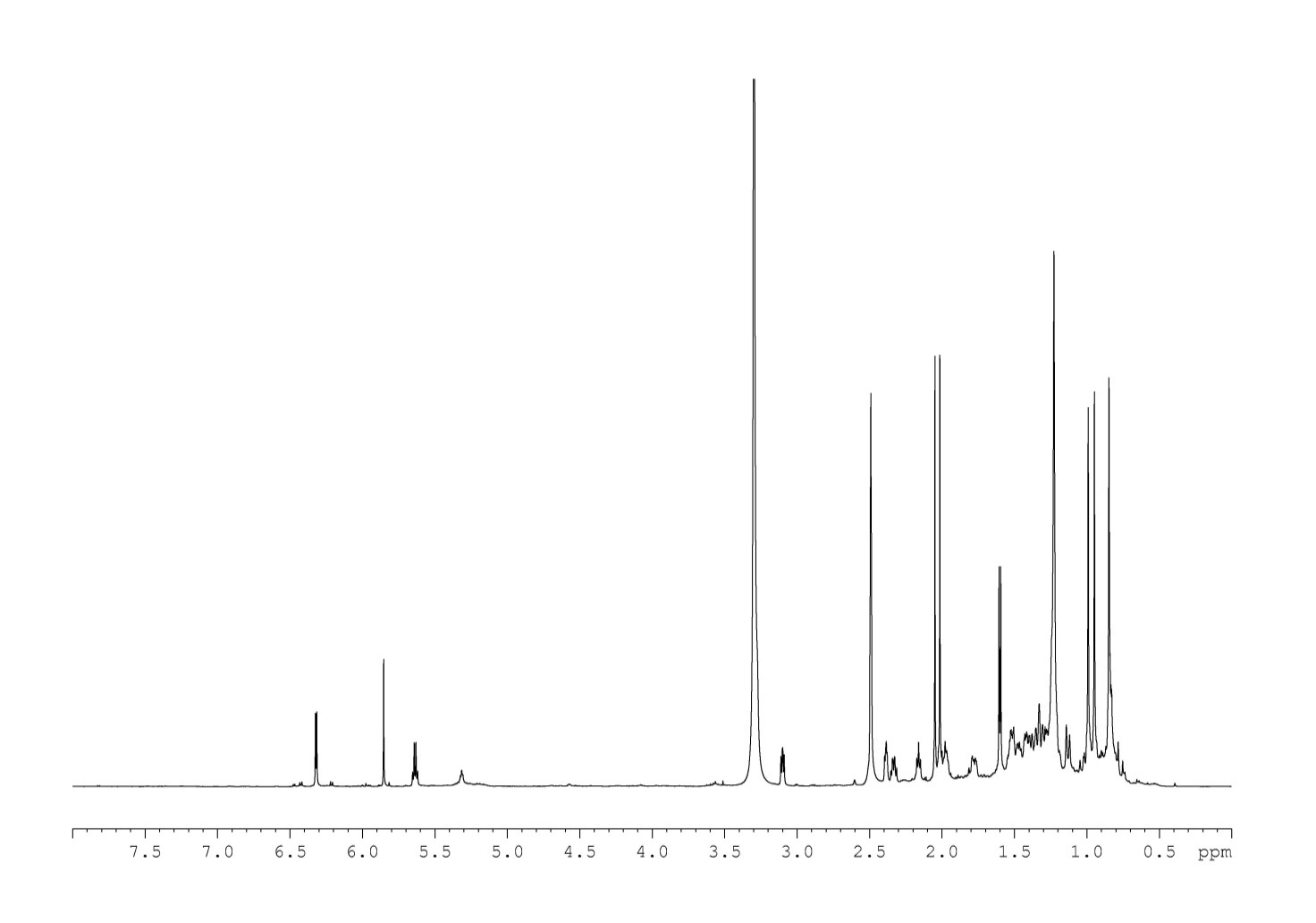


**Figure S4.** ^1^H-NMR spectrum of *Dendrilla membranosa* SPE-fraction D (600 MHz, DMSO-*d*_6_).


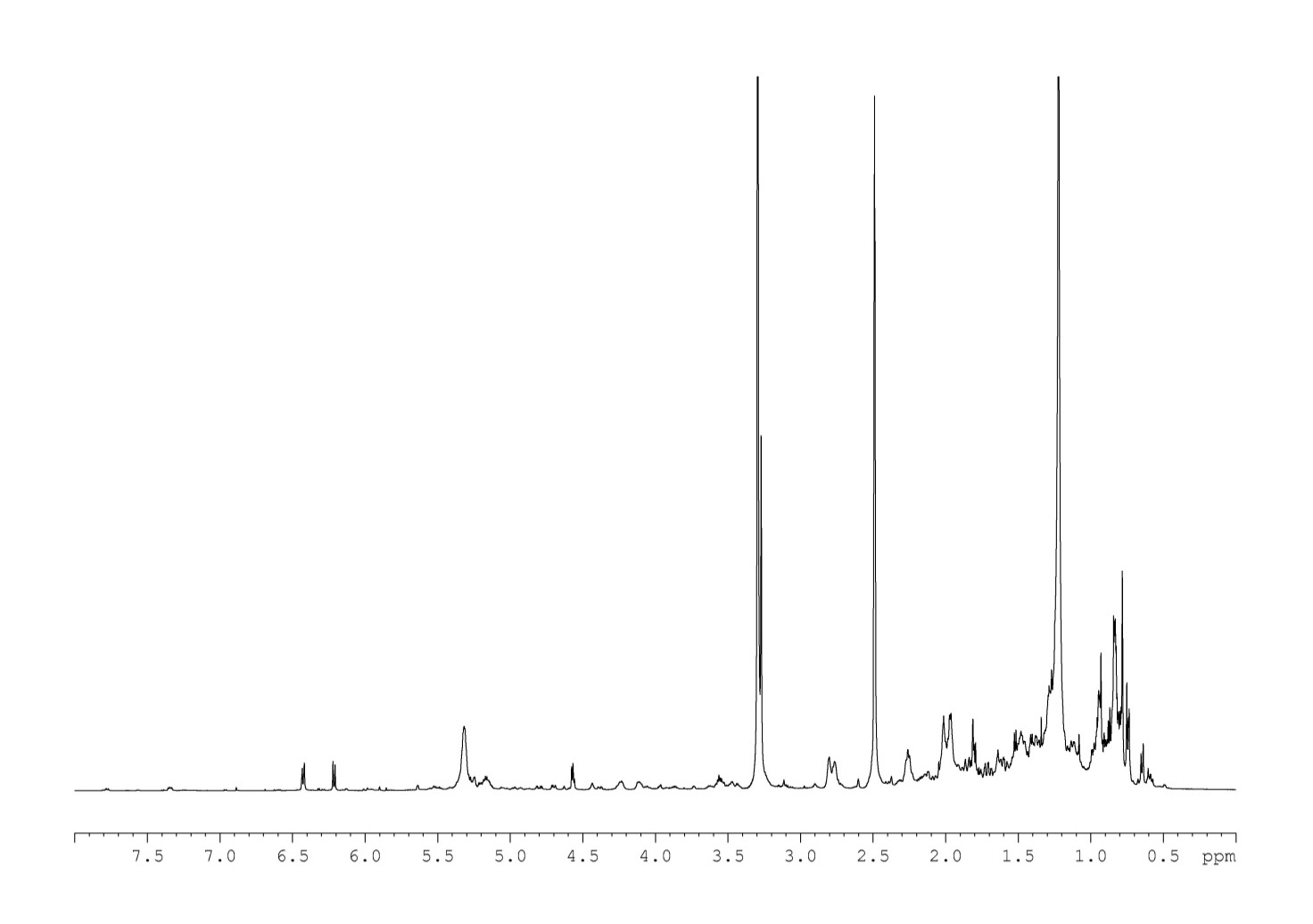


**Figure S5.** ^1^H-NMR spectrum of *Dendrilla membranosa* SPE-fraction E (600 MHz,
DMSO-*d*_6_).


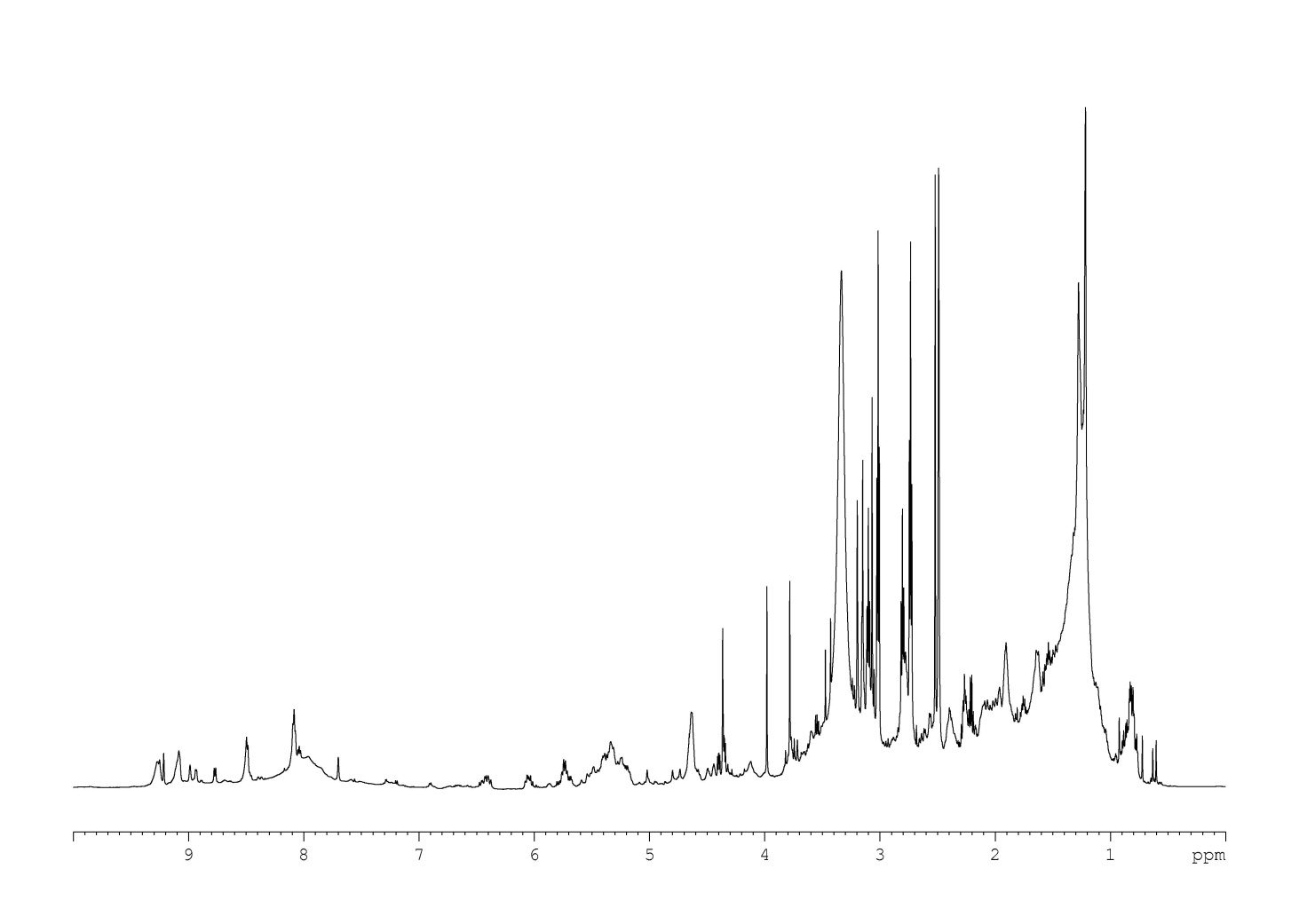


**Figure S6.** ^1^H-NMR spectrum of *Reniera sarai* extract (600 MHz, DMSO-*d*_6_).


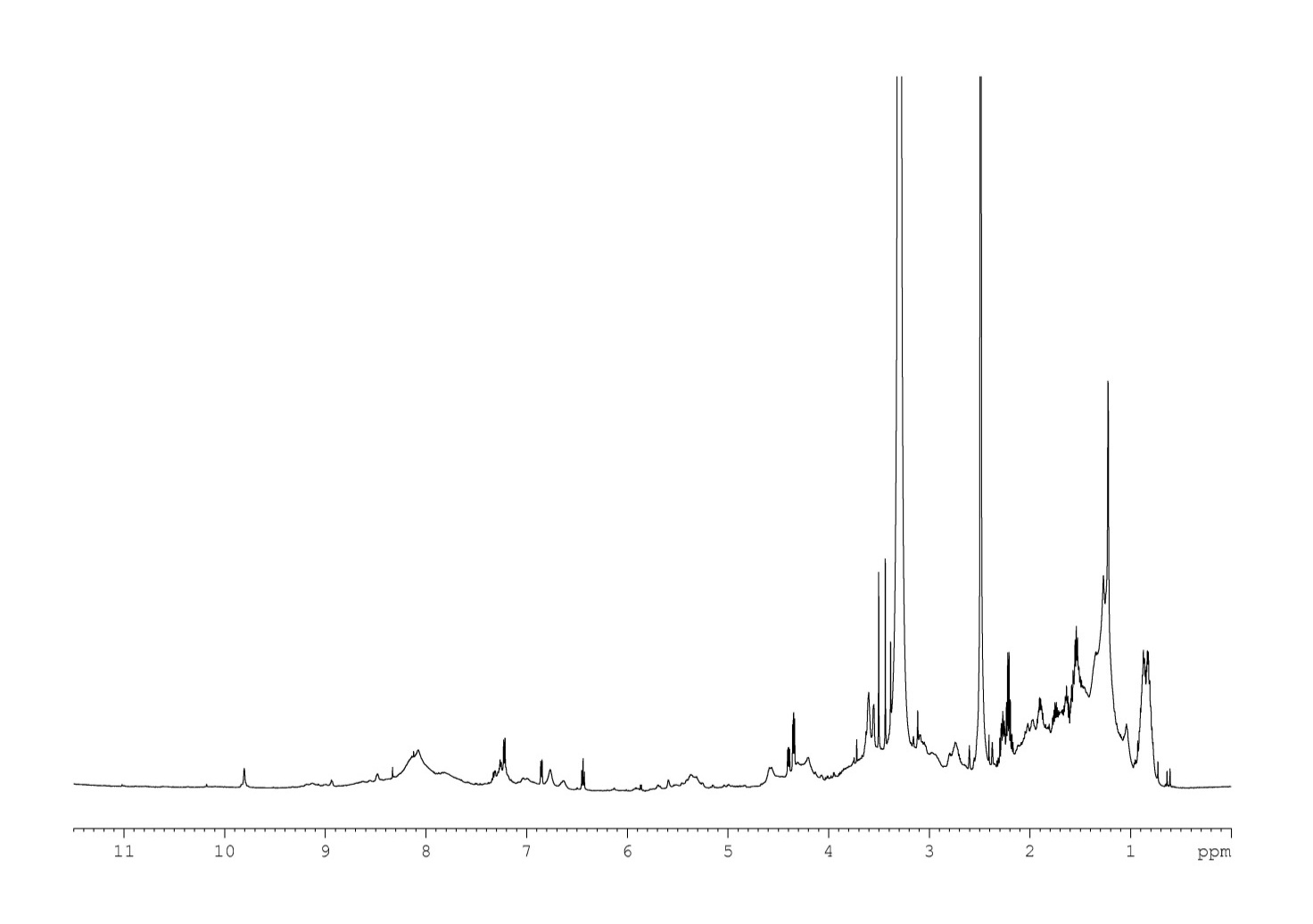


**Figure S7.** ^1^H-NMR spectrum of *Reniera sarai* SPE-fraction B (600 MHz, DMSO-*d*_6_).


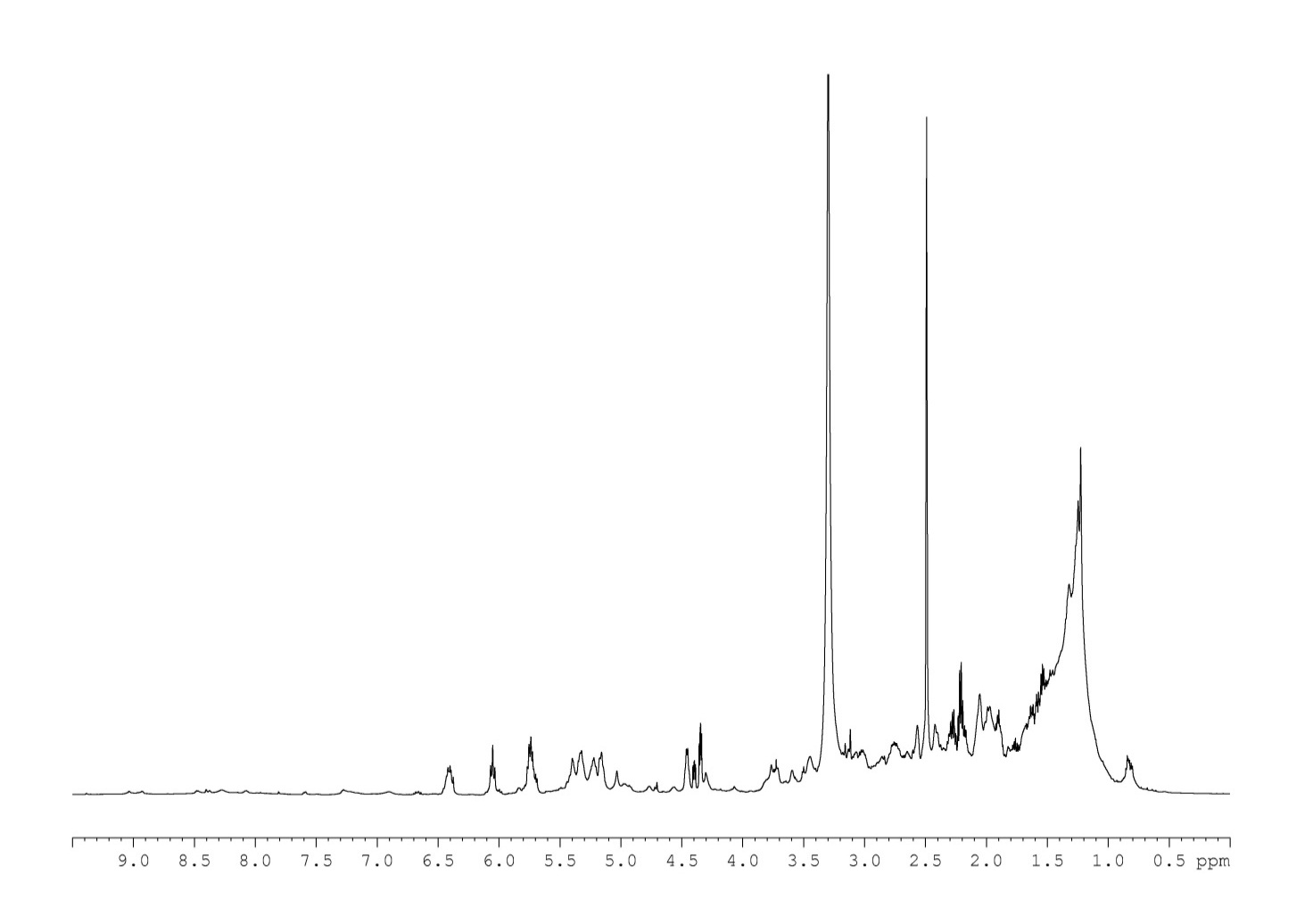


**Figure S8.** ^1^H-NMR spectrum of *Reniera sarai* SPE-fraction C (600 MHz, DMSO-*d*_6_).


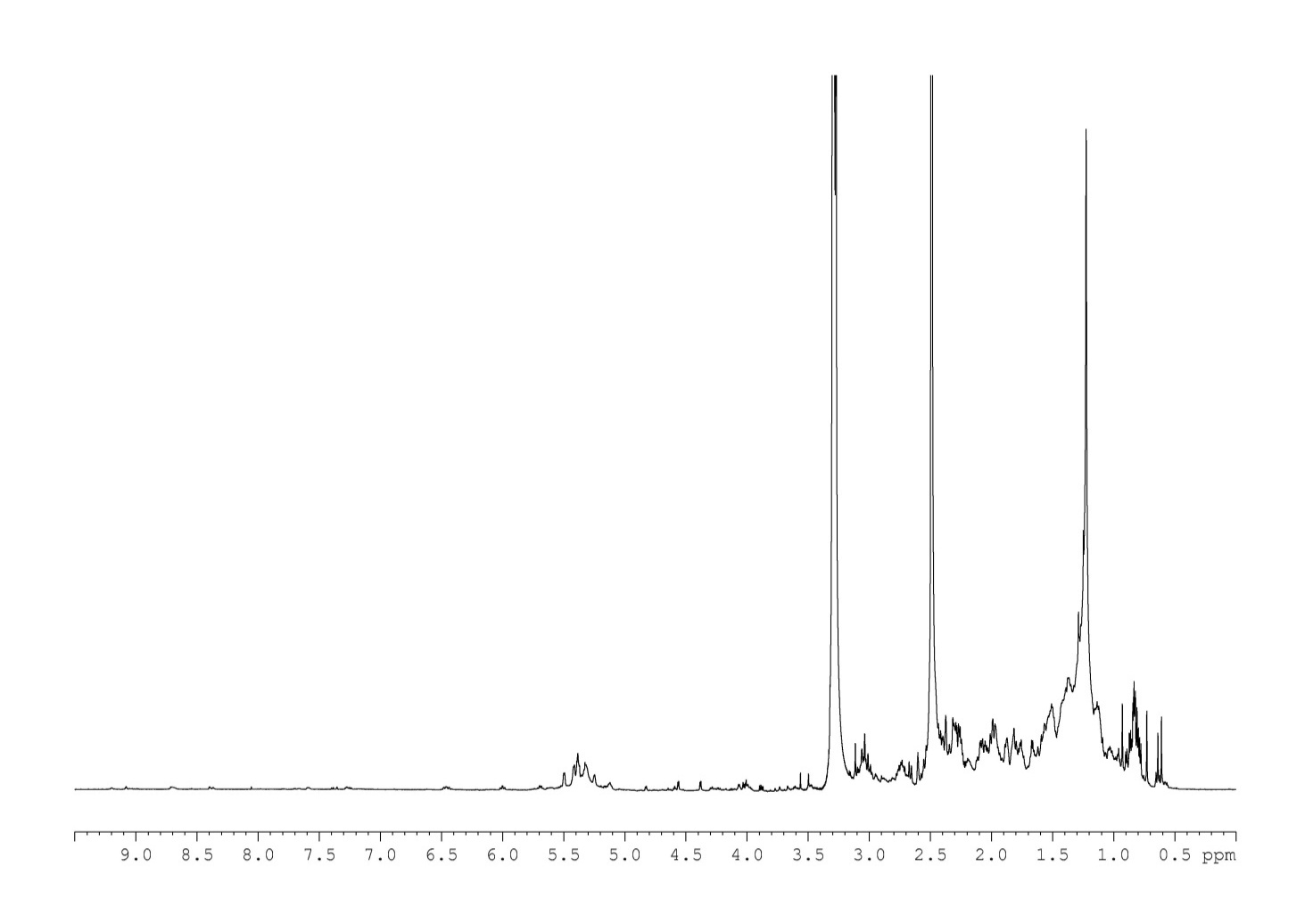


**Figure S9.** ^1^H-NMR spectrum of *Reniera sarai* SPE-fraction D (600 MHz, DMSO-*d*_6_).


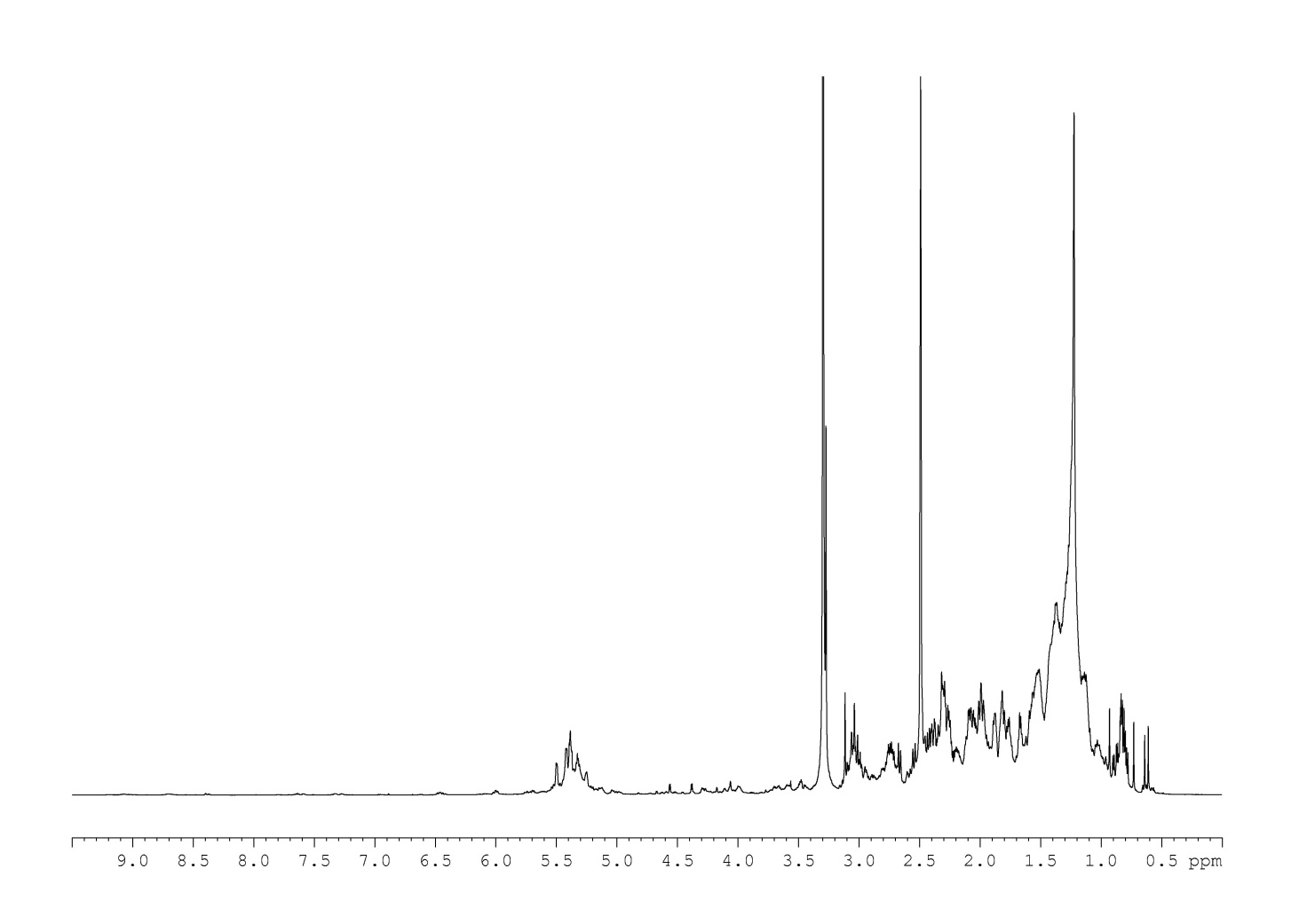


**Figure S10.** ^1^H-NMR spectrum of *Reniera sarai* SPE-fraction E (600 MHz, DMSO-*d*_6_).


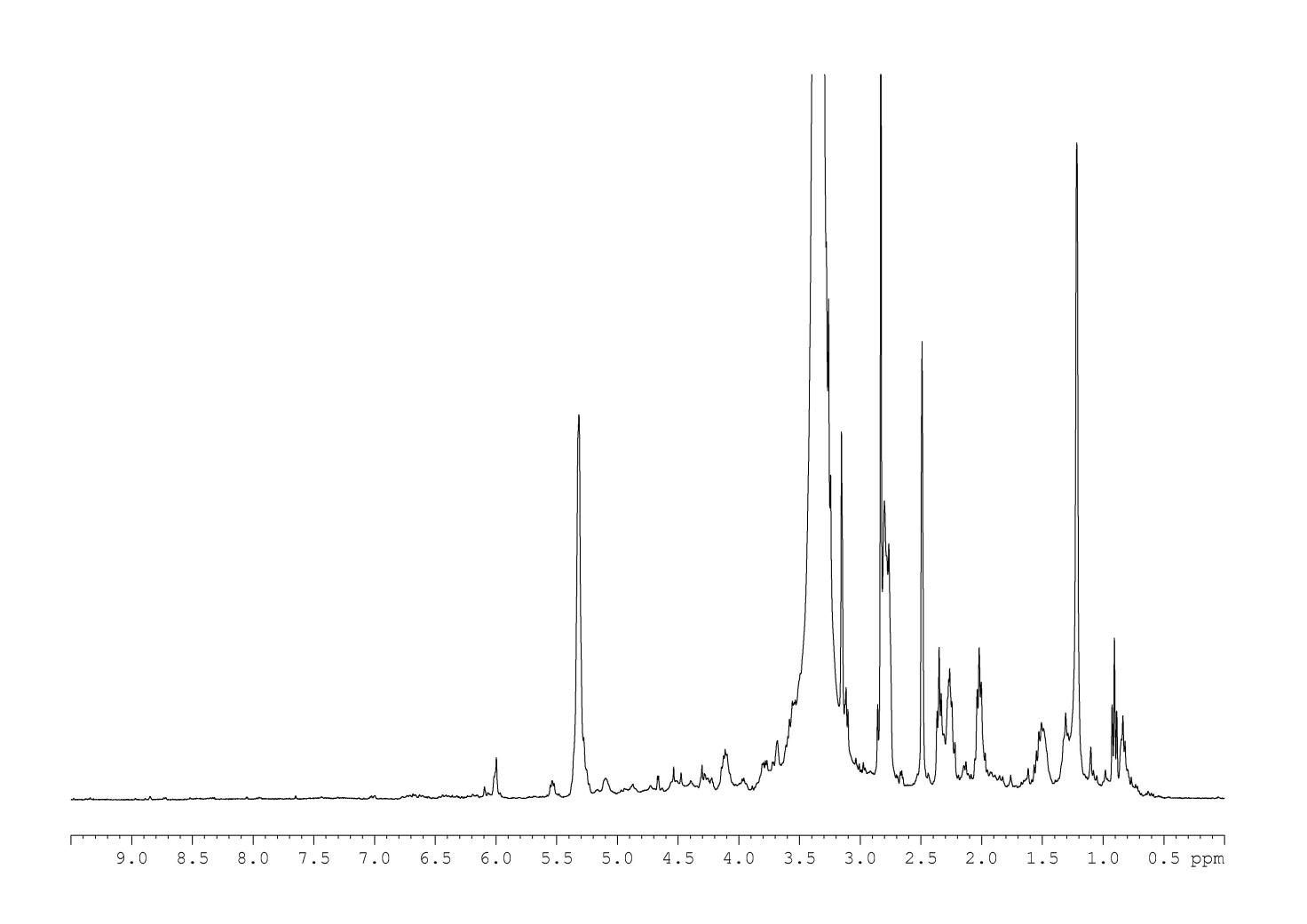


**Figure S11.** ^1^H-NMR spectrum of *Amphidinium carterae* extract (600 MHz, DMSO-*d*_6_).


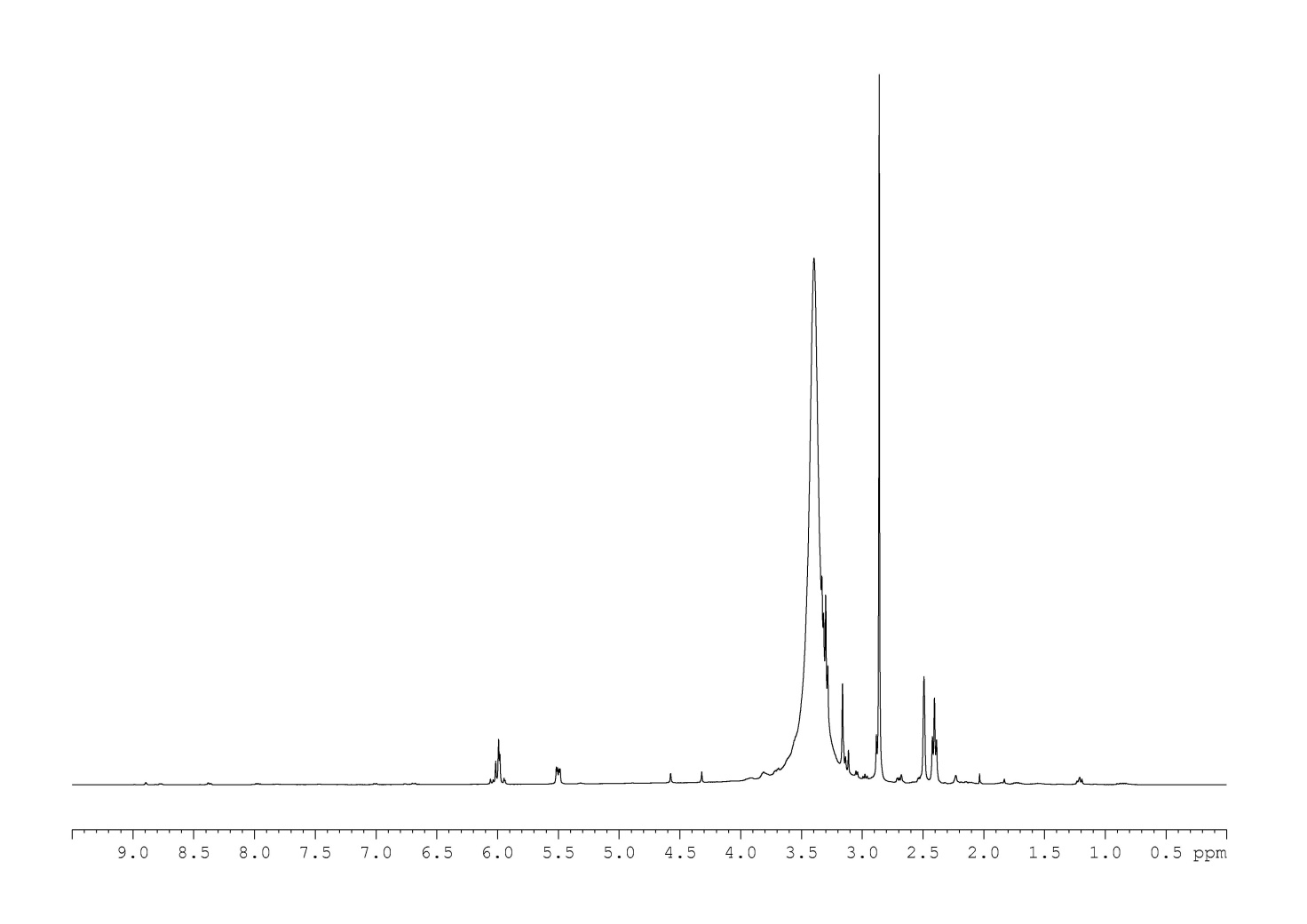


**Figure S12.** ^1^H-NMR spectrum of *Amphidinium carterae* SPE-fraction B (600 MHz, DMSO-*d*_6_).


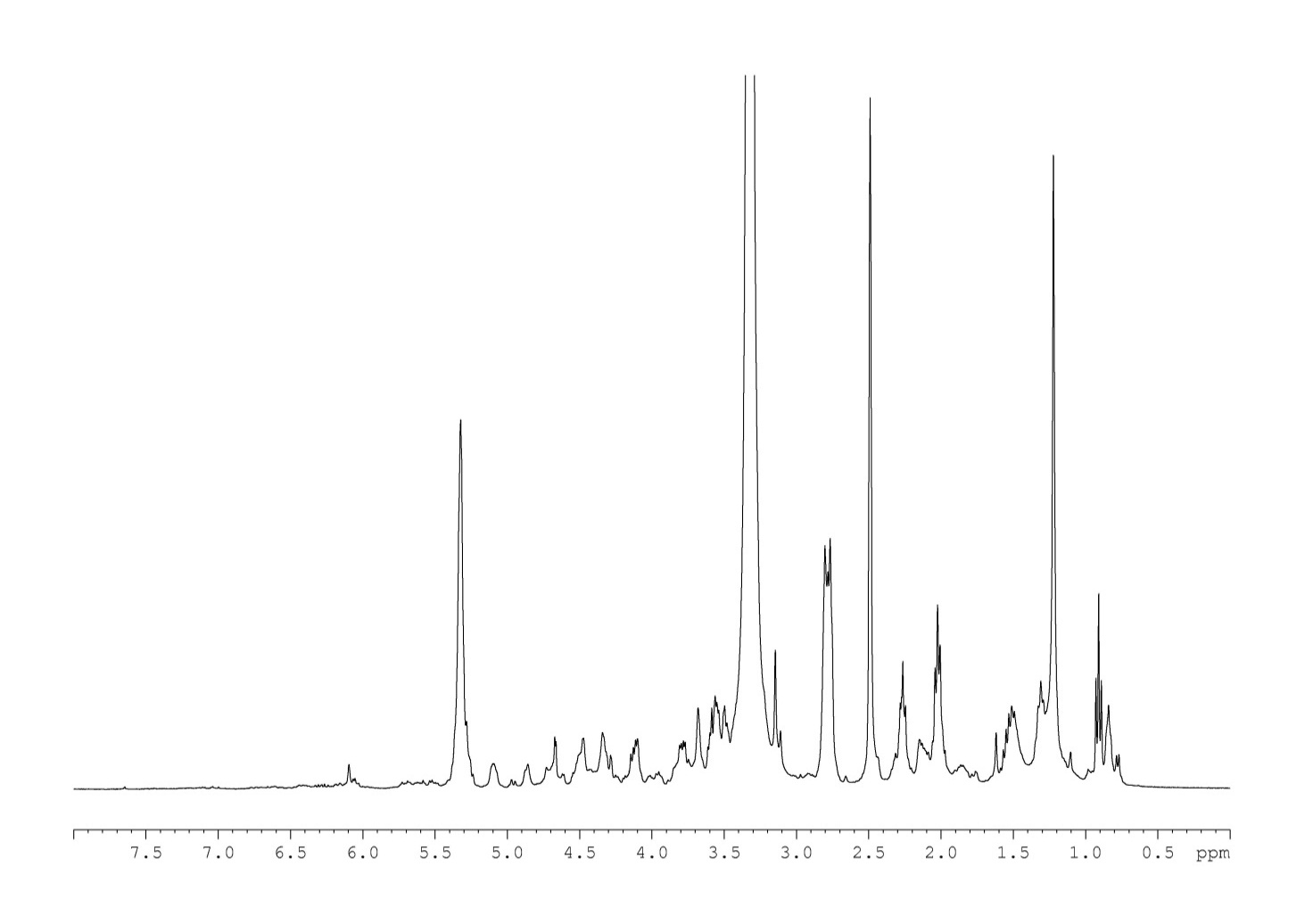


**Figure S13.** ^1^H-NMR spectrum of *Amphidinium carterae* SPE-fraction C (600 MHz, DMSO-*d*_6_).


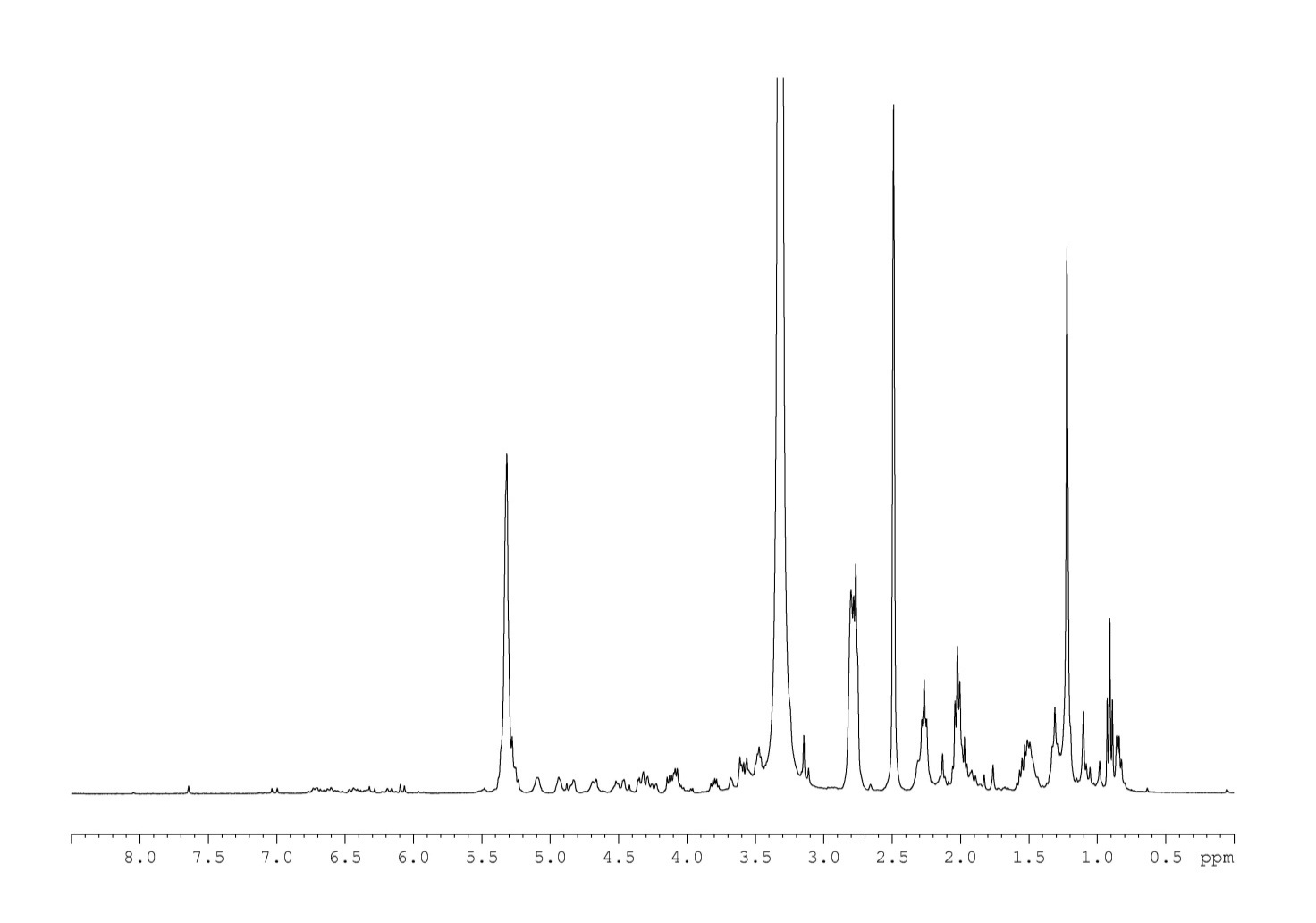


**Figure S14.** ^1^H-NMR spectrum of *Amphidinium carterae* SPE-fraction D (600 MHz, DMSO-*d*_6_).


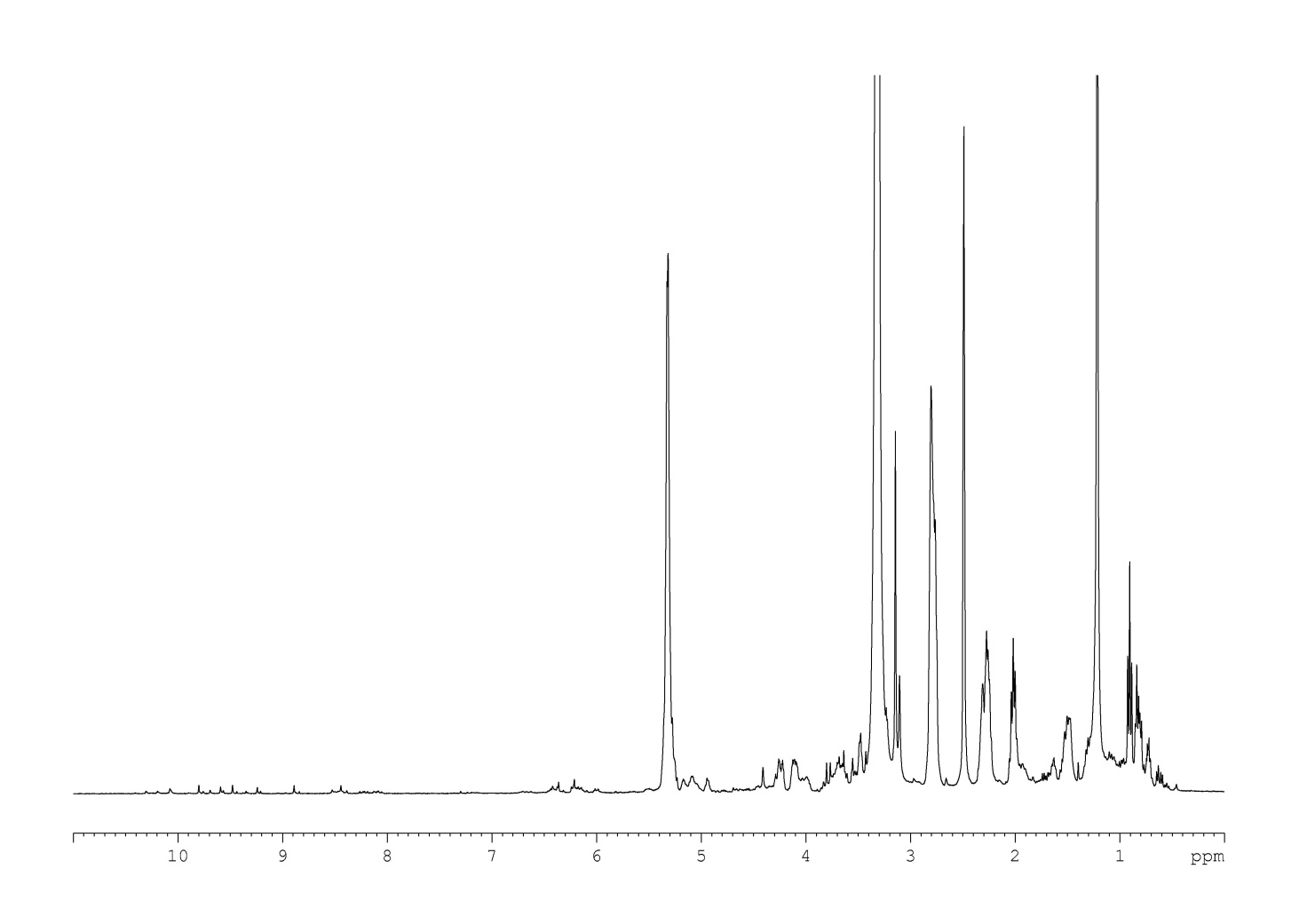


**Figure S15.** ^1^H-NMR spectrum of *Amphidinium carterae* SPE-fraction E (600 MHz, DMSO-*d*_6_).

**Figure S16.** LC-MS ESI^+^ of *Dendrilla membranosa* extract (column Phenomenex Luna C18 150 mm × 2 mm, 5 μm, linear gradient H_2_0:MeOH from 65% to 100 % of MeOH in 20 min and then 40 min of only MeOH).

**Figure S17.** LC-MS ESI^+^ of *Dendrilla membranosa* SPE-fraction B (column Phenomenex Luna C18 150 mm × 2 mm, 5 μm, linear gradient H_2_0:MeOH from 65% to 100 % of MeOH in 20 min and then 40 min of only MeOH).

**Figure S18.** LC-MS ESI^+^ of *Dendrilla membranosa* SPE-fraction C (column Phenomenex Luna C18 150 mm × 2 mm, 5 μm, linear gradient H_2_0:MeOH from 65% to 100 % of MeOH in 20 min and then 40 min of only MeOH).

**Figure S19.** LC-MS ESI^+^ of *Dendrilla membranosa* SPE-fraction D (column Phenomenex Luna C18 150 mm × 2 mm, 5 μm, linear gradient H_2_0:MeOH from 65% to 100 % of MeOH in 20 min and then 40 min of only MeOH).

**Figure S20.** LC-MS ESI^+^ of *Dendrilla membranosa* SPE-fraction E (column Phenomenex Luna C18 150 mm × 2 mm, 5 μm, linear gradient H_2_0:MeOH from 65% to 100 % of MeOH in 20 min and then 40 min of only MeOH).

**Figure S21.** LC-MS ESI^+^ of *Reniera sarai* extract (column Phenomenex Luna C18
150 mm × 2 mm, 5 μm, linear gradient H_2_0:MeOH from 65% to 100 % of MeOH in 20 min and then 40 min of only MeOH).

**Figure S22.** LC-MS ESI^+^ of *Reniera sarai* SPE-fraction B (column Phenomenex Luna C18 150 mm × 2 mm, 5 μm, linear gradient H_2_0:MeOH from 65% to 100 % of MeOH in 20 min and then 40 min of only MeOH).

**Figure S23.** LC-MS ESI^+^ of *Reniera sarai* SPE-fraction C (column Phenomenex Luna C18 150 mm × 2 mm, 5 μm, linear gradient H_2_0:MeOH from 65% to 100 % of MeOH in 20 min and then 40 min of only MeOH).

**Figure S24.** LC-MS ESI^+^ of *Reniera sarai* SPE-fraction D (column Phenomenex Luna C18 150 mm × 2 mm, 5 μm, linear gradient H_2_0:MeOH from 65% to 100 % of MeOH in 20 min and then 40 min of only MeOH).

**Figure S25.** LC-MS ESI^+^ of *Reniera sarai* SPE-fraction E (column Phenomenex Luna C18
150 mm × 2 mm, 5 μm, linear gradient H_2_0:MeOH from 65% to 100 % of MeOH in 20 min and then 40 min of only MeOH).

**Figure S26.** LC-MS ESI^+^ of *Amphidinium carterae* extract (column Phenomenex Luna C18 150 mm × 2 mm, 5 μm, linear gradient H_2_0:MeOH from 65% to 100 % of MeOH in 20 min and then 40 min of only MeOH).

**Figure S27.** LC-MS ESI^+^ of *Amphidinium carterae* SPE-fraction B (column Phenomenex Luna C18 150 mm × 2 mm, 5 μm, linear gradient H_2_0:MeOH from 65% to 100 % of MeOH in 20 min and then 40 min of only MeOH).

**Figure S28.** LC-MS ESI^+^ of *Amphidinium carterae* SPE-fraction C (column Phenomenex Luna C18 150 mm × 2 mm, 5 μm, linear gradient H_2_0:MeOH from 65% to 100 % of MeOH in 20 min and then 40 min of only MeOH).

**Figure S29.** LC-MS ESI^+^ of *Amphidinium carterae* SPE-fraction D (column Phenomenex Luna C18 150 mm × 2 mm, 5 μm, linear gradient H_2_0:MeOH from 65% to 100 % of MeOH in 20 min and then 40 min of only MeOH).

**Figure S30.** LC-MS ESI^+^ of *Amphidinium carterae* SPE-fraction E (column Phenomenex Luna C18 150 mm × 2 mm, 5 μm, linear gradient H_2_0:MeOH from 65% to 100 % of MeOH in 20 min and then 40 min of only MeOH).

© 2015 by the authors; licensee MDPI, Basel, Switzerland. This article is an open access article distributed under the terms and conditions of the Creative Commons Attribution license (http://creativecommons.org/licenses/by/4.0/).
